# Increased incidence of human leptospirosis and the effect of temperature and precipitation, the Netherlands, 2005 to 2023

**DOI:** 10.2807/1560-7917.ES.2025.30.15.2400611

**Published:** 2025-04-17

**Authors:** Ilja Obels, Lapo Mughini-Gras, Miriam Maas, Diederik Brandwagt, Nikita van den Berge, Daan W Notermans, Eelco Franz, Erika van Elzakker, Roan Pijnacker

**Affiliations:** 1 Centre for Infectious Diseases Control, National Institute for Public Health and the Environment (RIVM), Bilthoven, the Netherlands; 2 Institute for Risk Assessment Sciences, Utrecht University, Utrecht, the Netherlands; 3 Expertise Centre for Leptospirosis, Department of Medical Microbiology and Infection Prevention, Amsterdam University Medical Center (UMC), Amsterdam, the Netherlands

**Keywords:** Leptospirosis, climate change, water-borne infectious diseases, incidence

## Abstract

**Background:**

The incidence of leptospirosis, a zoonotic infection transmitted mainly by rodents, has increased in humans over the past decade in the Netherlands. Previous studies, mostly from countries with tropical climates, suggest that temperature and rainfall influence leptospirosis incidence.

**Aim:**

We aimed to identify factors that could explain the increasing leptospirosis incidence in the Netherlands, including temperature and precipitation.

**Methods:**

Epidemiological data of leptospirosis cases notified from 2005 to 2023 to the national surveillance system were analysed to identify changes over the years. Negative binomial regression models were used to assess associations between weather variables and leptospirosis incidence.

**Results:**

From 2005 to 2023, 1,164 cases were notified. The annual number of cases increased 2.7-fold in the period of 2019–2023 compared with 2005–2009, and the number of autochthonous cases 4.1-fold. Data from 1,158 cases were included in the analyses, and 596 (51.5%) of these cases were autochthonous. Most cases were male (n = 927; 80.1%), needed hospital treatment (n = 861; 74.4%) and acquired the infection through contact with surface water and/or soil (n = 611, 72.6%). Higher mean winter (incidence rate ratio (IRR) = 1.27; 95% confidence interval (CI): 1.18–1.36) and summer (IRR = 1.38; 95% CI: 1.18–1.61) temperatures were significantly associated with increased leptospirosis incidence.

**Conclusion:**

Leptospirosis incidence has increased over the past decades and may continue to increase due to climate change. Prevention should aim at advising the appropriate preventive measures to avoid exposure to *Leptospira* and increasing awareness about leptospirosis among clinicians to allow for timely diagnosis and treatment.

**Key public health message**

**What did you want to address in this study and why?**

Leptospirosis is an infectious disease caused by bacteria of *Leptospira.* These bacteria are spread by rodents. In the Netherlands, infections of *Leptospira* have been rising over the past decade. We investigated what might be the cause of this increase, including climate factors like temperature and rainfall. We also looked into how cases are getting infected, such as through contact with surface water or animals.

**What have we learnt from this study?**

From 2005 to 2023, leptospirosis was diagnosed in 1,164 persons in the Netherlands. The number of leptospirosis cases increased over time. Most of these patients were male (n = 927) and needed hospital treatment (n = 861). Half of the cases (n = 596) had been infected in the Netherlands. Higher temperatures were linked to more cases. Contact with contaminated surface water or soil was the most common likely source of the infection.

**What are the implications of your findings for public health?**

The number of leptospirosis cases may further increase due to climate change. Increased awareness among physicians on leptospirosis and public health campaigns about risks, like those from recreational water activities, are needed. Good hygiene practices are needed for workers on farms and in sewage management.

## Introduction

Leptospirosis is a zoonotic infection caused by pathogenic species of bacteria of the genus *Leptospira*. Rodents, dogs, cattle and other mammals can serve as hosts that shed *Leptospira* via their urine when infected [1,2]. Human infection occurs via exposure to urine or other body fluids of infected carrier mammals, either directly or indirectly via contact with contaminated soil or water [1,2]. *Leptospira* bacteria enter the body via cuts and abrasions of the skin or via the mucous membranes of the conjunctivae or the oral cavity [1]. The incubation period is most often 7–12 days but can range from 3 to 30 days [1]. Infections are usually self-limiting, with febrile illness and other unspecific symptoms, however, severe and potentially lethal illness may occur in a minority of cases [1,3].

Although regions with a tropical climate have the highest leptospirosis incidence, with most cases reported from South-eastern Asia, South America and Sub-Saharan Africa, leptospirosis is also endemic in temperate regions, such as Europe [3]. In a recent report of the European Environment Agency (EEA) on climate change as a threat to health, leptospirosis was listed as one of the infectious diseases sensitive to climatic and weather factors [4]. Indeed, previous studies have suggested that temperature and rainfall are significantly associated with notification rates of leptospirosis [5-8]. While most of these studies were performed in countries with tropical climates, climate change is likely to increase the risk of leptospirosis in both tropical and temperate climates due to higher temperatures and heavy rainfall, but also due to climate hazards leading to human displacement or impaired sewage systems [9]. Notified incidence of leptospirosis in Europe increased with an average of 5% per year from 2010 to 2021 [3,10], and the Netherlands had one of the highest notification rates in Europe, particularly since 2014 [10,11]. Possible explanations for the increase were an increasing popularity of water-based activities [10,12,13], changes in diagnostic practices or notification criteria and changing weather conditions [10]. In the Netherlands, an increase in the number of leptospirosis cases was observed in 2014, considered to be caused by a mild winter followed by an exceptionally warm year [14].

The aim of this study was to identify epidemiological factors that could explain the increase in the number of autochthonous and imported leptospirosis cases in the Netherlands, including source and country of infection. For autochthonous cases, we determined their annual and weekly association with temperature and precipitation.

## Methods

### Data collection

Leptospirosis has been a notifiable disease in the Netherlands since 1928 [15]. Laboratories and physicians have to mandatorily notify the municipal health service (MHS) when they detect a case of leptospirosis (Box). The MHS collects case characteristics, performs source tracing through patient interviews of all cases and reports each case fitting the case definition to the national surveillance database of the National Institute for Public Health and the Environment (RIVM) [16]. The case definition for notification of a leptospirosis case is presented in Box.

Box **Case definition for notification of a leptospirosis case, the Netherlands**

**Clinical criteria:**

• A person with fever and at least two of the following symptoms or conditions: headache, myalgia, chills, diarrhoea and/or vomiting, conjunctival suffosion, bleeding in skin and mucous membranes, rash, jaundice, myocarditis, meningitis, renal impairment, pulmonary haemorrhagic symptoms

OR

**Epidemiological criteria:**

• A high-risk contact with an infected animal or contaminated area^a^

AND

**Laboratory criteria:**

At least one of these diagnostic criteria:

• Detection of *Leptospira* by culture or PCR

OR

• Serological response (MAT or IgM ELISA)

OR

• Positive rapid diagnostic test which refers to a lateral flow immunochromatographic test for detection of *Leptospira*-specific IgM antibodies [21].

MAT: microscopic agglutination test.
^a^ High-risk contact as judged by the municipal health service (MHS).

We used surveillance reports from the RIVM national surveillance database to obtain leptospirosis notifications from 2005 to 2023. We collected information on the date of onset of symptoms, age, sex, country of infection, diagnostic test, hospitalisation status and whether an infection was considered occupational. Furthermore, we extracted the most likely source(s) of infection as reported by the MHS. Since 2014, the likely sources have been categorised followingly: surface water or soil, animal contact, other and unknown, with unknown consisting of cases with multiple likely sources of infection and those for which no source could be identified. Since 2014, the national surveillance database has been linked to the laboratory database of the National Leptospirosis Reference Centre (NRL), Amsterdam University Medical Center (UMC). In 2023, the NRL was named the Expertise Centre for Leptospirosis (XCL). For data completeness, we aimed to identify cases that were diagnosed but not notified, based on postal code, sex and year of birth [14]. Data from 2005 to 2023 on temperature and precipitation in De Bilt, a weather station located in the middle of the Netherlands, were obtained for from the Royal Meteorological Institute (KNMI) [17]. We obtained the number of inhabitants of the Netherlands per year, sex and age group from the Statistics Netherlands (CBS) [18].

For the cases that did not have information on the date of onset of symptoms, the date of diagnosis was used, and if not available, the date of notification. If these dates were missing, the cases were excluded. Based on source tracing by the MHS, cases that most likely acquired the infection in the Netherlands were classified as autochthonous, and those that acquired the infection abroad as imported. The most likely country of infection was determined by the MHS and could also be unknown.

### Weather variables

We computed the following variables on temperature in winter (December–February): mean temperature, minimum temperature (the lowest monthly mean minimum temperature in the winter months) and mean minimum temperature (the mean of the monthly mean minimum temperatures in the winter months). Additionally, we computed the mean temperature in summer (June–September). Moreover, yearly mean temperature was obtained. The variables on precipitation included the following: cumulative yearly and weekly precipitation (in 10 mm), cumulative yearly precipitation in June–October and June–September (in 10 mm). Additionally, we obtained the weekly mean, minimum and maximum temperature.

### Statistical analysis

Descriptive statistics were stratified by autochthonous and imported infections, as well as by the year of infection. To determine whether there was a trend over time in age, sex, diagnostic test, hospital admissions, country of infection, region of infection (South-eastern Asia, Central America and Caribbean, Europe, South America, other and unknown), source of infection and occupational infections, we performed the Jonckheere-Terpstra test for trend [19,20].

To test for the association between the number of autochthonous cases and weather variables from 2005 to 2023, two negative binomial regression models were built, including the available information on (estimated) date of infection, age and sex. The first model associated the number of notified cases with temperature and precipitation by year. The second model associated the number of cases with temperature and precipitation by week June–October, the months in which most infections occurred. In this model, a 2-week lag was applied on the weather variables, which was chosen based on Pearson correlation coefficients of the correlation between lag variables varying from 1 to 4 weeks and the number of weekly leptospirosis cases and incubation time (maximum of 4 weeks). The Pearson correlation coefficients are presented in Supplementary Table 1. Univariable negative binomial regression was performed to assess the association between weather variables and the number of leptospirosis cases. In all models, population size per year was included as offset to be able to calculate incidence rate ratios (IRRs). All variables that were significant in univariable regression were entered in multivariable models that were adjusted for age, sex and for the COVID-19 pandemic years of 2020 and 2021. Collinearity between all variables was assessed by Pearson correlation coefficients, as presented in Supplementary Table 2 and 3. If the correlation coefficients were > 0.5 or when a strong correlation was expected based on previous knowledge, only the variable with the lowest p value in univariable regression was included in the multivariable model. In sensitivity analyses, we ran negative binomial regression models to assess whether there were significant interactions between temperature variables and age and sex. The rationale was that temperature-dependent behaviour in humans that is prone to *Leptospira* infection, such as swimming in surface water, may be age- and sex-dependent as well.

Results from negative binomial regression models were expressed as IRRs with corresponding 95% confidence intervals (CIs). The chosen level of significance for all analyses was p < 0.05. The analyses were performed in R statistical software, version 4.4.0 (https://www.r-project.org/).

## Results

In total, 1,164 leptospirosis cases were notified in the Netherlands from January 2005 to December 2023. Six cases were excluded from our analyses due to missing data on the year of infection. Of the included cases, 596 (51.5%) were considered autochthonous infections, 507 (43.8%) were imported (travel-related) and for 55 (4.7%) cases, the country of infection was unknown.

From 2005 to 2013, an average of 35 leptospirosis cases per year were notified, with a sudden increase to 90 cases in 2014 (Figure). After a gradual decrease in the following years, the number of cases increased to 124 in 2019 and has remained at this level since, although the number of imported cases was lower in 2020 and 2021. The annual mean number of cases was 2.7-fold higher in the period 2019–2023 (mean = 92; range: 58–124) than 2005–2009 (mean = 34; range: 26–46). The increase was 4.1-fold for autochthonous cases in the period 2019–2023 (mean = 57; range: 49–74) compared with 2005–2009 (mean = 14; range: 11–22). The incidence ranged from 0.05 per 100,000 population in 2013 to 0.42 per 100,000 population in 2023.

**Figure:**
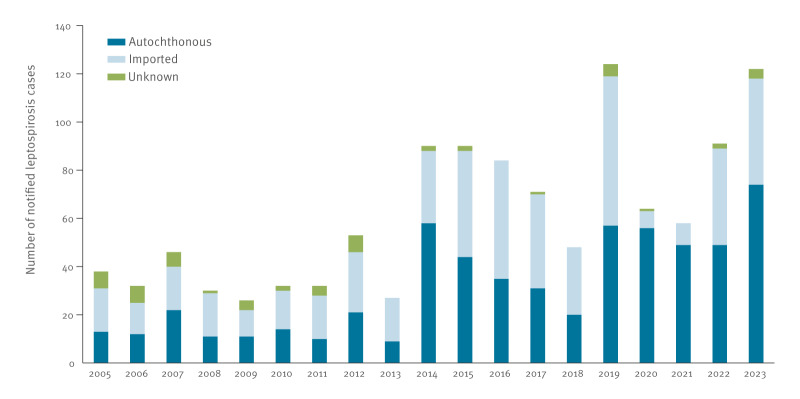
Number of notified leptospirosis cases, by country of infection, the Netherlands, 2005–2023 (n = 1,158)

### Autochthonous cases

Of the 596 autochthonous cases, 502 (84.2%) were male and 232 (38.9%) were aged 40–59 years (Table 1). Hospital admission was required for 511 (85.7%) cases. Culture and/or PCR (n = 228, 38.3%) and serology (n = 235, 39.4%) were the most applied diagnostic tests. Data on the likely source of infection were available for 2014–2023. Most infections (n = 337, 71.2%) were acquired through contact with contaminated surface water or soil, 49 (10.4%) cases acquired the infection by direct contact with infected animals and for 86 (18.2%) cases, the most likely source of infection was unknown. For 90 (19.0%) cases, the infection was considered occupational.

**Table 1:** Characteristics of notified leptospirosis cases, by country of infection, the Netherlands, 2005–2023 (n = 1,158)

Characteristics	Total n = 1,158		Autochthonous n = 596		Imported n = 507		Unknown n = 55	
	n	%	n	%	n	%	n	%
Sex								
Female	220	19.0	93	15.6	118	23.3	9	16.4
Male	927	80.1	502	84.2	389	76.7	36	65.5
Unknown	11	0.9	1	0.2	0	0.0	10	18.2
Age group (years)								
0–4	2	0.2	2	0.3	0	0.0	0	0.0
5–17	125	10.8	64	10.7	56	11.0	5	9.1
18–39	466	40.2	156	26.2	289	57.0	21	38.2
40–59	373	32.2	232	38.9	129	25.4	12	21.8
≥ 60	181	15.6	141	23.7	33	6.5	7	12.7
Unknown	11	0.9	1	0.2	0	0.0	10	18.2
Diagnostic test								
PCR and/or culture	428	37.0	228	38.3	196	38.7	4	7.3
Serology	485	41.9	235	39.4	239	47.1	11	20.0
Rapid diagnostic test	49	4.2	35	5.9	13	2.6	1	1.8
Multiple tests	98	8.5	65	10.9	33	6.5	0	0.0
Unknown	98	8.5	33	5.5	26	5.1	39	70.9
Hospital admission								
Yes	861	74.4	511	85.7	335	66.1	15	27.3
No	203	17.5	63	10.6	138	27.2	2	3.6
Unknown	94	8.1	22	3.7	34	6.7	38	69.1
Region of infection^a^								
South-eastern Asia	NA		NA		303	59.8	NA	
Central America and the Caribbean					79	15.6		
Europe					62	12.2		
South America					38	7.5		
Other					20	3.9		
Unknown					5	1.0		
Source of infection (n = 842)^b^								
Surface water or soil	611	72.6	337	71.2	272	77.3	2	11.8
Animal contact	52	6.2	49	10.4	3	0.9	0	0.0
Other	1	0.1	1	0.2	0	0.0	0	0.0
Unknown	178	21.1	86	18.2	77	21.9	15	88.2
Occupational infection (n = 842)^b^								
Yes	98	11.6	90	19.0	8	2.3	0	0.0
No	643	76.4	323	68.3	317	90.1	3	17.6
Unknown	101	12.0	60	12.7	27	7.7	14	82.4

NA:  not applicable.

^a^ Only described for imported cases.

^b^ Information on the source of infection and occupational infection were available for cases notified 2014–2023.

Most of the epidemiological characteristics of autochthonous cases were similar over the study period, presented in Supplementary Table 4. However, the proportion of cases diagnosed using PCR and/or culture increased compared with serology (p < 0.01). Furthermore, there was a small increase in the proportion of hospitalised cases (p = 0.03).

### Imported cases

Most of the 507 imported cases were male (n = 389, 76.7%), and 289 (57.0%) were aged 18–39 years (Table 1). Hospitalisation was necessary for 335 (66.1%) cases, and 239 (47.1%) were diagnosed by serology. The geographical regions where most imported cases had been infected were South-eastern Asia (n = 303, 59.8%), mainly Thailand (n = 190, 37.5%), Central America and the Caribbean (n = 79, 15.6%) and Europe (n = 62, 12.2%). Most cases (n = 272, 77.3%) were transmitted through contact with contaminated surface water or soil. However, for 77 (21.9%) cases the most likely source of infection was unknown. Only 8 (2.3%) infections were occupational.

As for autochthonous cases, the reported diagnostic test changed over time from serology towards PCR and/or culture (p < 0.01), presented in Supplementary Table 5. Additionally, the hospitalisation rate slightly decreased (p < 0.01).

### Leptospirosis incidence and weather on year-level

The negative binomial regression model on factors associated with the yearly leptospirosis incidence of autochthonous cases included 585 cases, as 10 cases with missing (estimated) date of onset of symptoms and one with missing age were excluded. In Supplementary Figure 1 and 2, the weather variables are shown over time. In univariable regression analysis, all temperature variables included were significantly associated with increased leptospirosis incidence: higher mean minimum temperature during winter (IRR = 1.31; 95% CI: 1.15–1.49), higher mean (IRR = 1.35; 95% CI: 1.19–1.52) and minimum (IRR = 1.21; 95% CI: 1.12–1.32) temperature during winter, higher mean temperature during the whole year (IRR = 1.81; 95% CI: 1.38–2.36) and higher mean temperature during the summer (IRR = 1.60; 95% CI: 1.18–2.17) (Table 2). Furthermore, annual cumulative precipitation (IRR = 1.02; 95% CI: 1.01–1.04) and male sex (IRR = 5.02; 95% CI 3.69–6.88) were significantly associated with increased leptospirosis incidence. Incidences among persons aged 0–4 years (IRR = 0.05; 95% CI: 0.01–0.16) and 5–17 years (IRR = 0.50; 95% CI: 0.29–0.86) were lower compared with those aged 40–59 years.

**Table 2:** Results of univariable and multivariable negative binomial regression models for factors associated with annual leptospirosis incidence, the Netherlands, 2005–2023 (n = 585)

Variables	Crude IRR	95% CI	Adjusted IRR^a^	95% CI
Weather				
Mean minimum temperature winter (°C)^b^	1.31	1.15–1.49	NT	
Mean temperature winter (°C)^b^	1.35	1.19–1.52	1.27	1.18–1.36
Minimum temperature winter (°C)^b^	1.21	1.12–1.32	NT	
Mean temperature year (°C)	1.81	1.38–2.36	NT	
Mean temperature summer (°C)^c^	1.60	1.18–2.17	1.38	1.18–1.61
Cumulative precipitation per year (10 mm)	1.02	1.01–1.04	1.00	1.00–1.00
Cumulative precipitation June–October (10 mm)	1.01	0.98–1.03	NA	
Cumulative precipitation June–September (10 mm)	0.99	0.96–1.02	NA	
Age group (years)				
0–4	0.05	0.01–0.16	0.05	0.01–0.14
5–17	0.50	0.29–0.86	0.50	0.36–0.68
18–39	0.65	0.39–1.08	0.65	0.51–0.83
40–59	Reference			
≥ 60	0.71	0.43–1.19	0.70	0.54–0.90
Sex				
Female	Reference			
Male	5.02	3.69–6.88	5.53	4.38–7.06

CI: confidence interval; IRR: incidence rate ratio; NA: not applicable; NT: not tested.

^a^ The multivariable negative binomial regression model was adjusted for whether a case was infected in a COVID-19 pandemic year of 2020 or 2021.

^b^ December, January and February were considered winter months. The winter months of a year were the months January and February of that specific year and the month December of the previous year.

^c^ June, July, August and September were considered summer months.

In the multivariable negative binomial regression model on leptospirosis incidence per year, mean winter temperature (IRR = 1.29; 95% CI: 1.20–1.38), mean summer temperature (IRR = 1.40; 95% CI 1.20–1.63) and male sex (IRR = 5.55; 95% CI: 4.38–7.10) significantly increased leptospirosis incidence per year (Table 2). Incidence was lower in age groups 0–4 years (IRR = 0.05; 95% CI: 0.01–0.14), 5–17 years (IRR = 0.50; 95% CI: 0.37–0.68), 18–39 years (IRR = 0.65; 95% CI: 0.51–0.83) and ≥ 60 years (IRR = 0.70; 95% CI: 0.55–0.90) compared with 40–59 years.

### Leptospirosis incidence and weather on week level

In negative binomial regression on factors associated with weekly incidence of leptospirosis, we included 443 autochthonous cases with a date of symptom onset June–October. In univariable regression analysis, higher mean temperature (IRR = 1.05; 95% CI: 1.02–1.09), higher minimum temperature (IRR = 1.06; 95% CI: 1.02–1.10), higher maximum temperature (IRR = 1.04; 95% CI: 1.01–1.07) and male sex (IRR = 5.78; 95% CI: 4.45–7.63) were significantly associated with increased leptospirosis incidence June–October (Table 3). In addition, the incidence was significantly lower among persons aged 0–4 years (IRR = 0.06; 95% CI:0.01–0.19), 5–17 years (IRR = 0.54; 95% CI: 0.38–0.75), 18–39 years (IRR = 0.67; 95% CI: 0.52–0.88) and ≥ 60 years (IRR = 0.70; 95% CI: 0.53–0.92) than among those aged 40–59 years.

**Table 3:** Results of univariable and multivariable negative binomial regression models of factors associated with weekly incidence of autochthonous leptospirosis June–October, the Netherlands, 2005–2023 (n = 443)

Characteristics	Univariable IRR		95% CI	Multivariable IRR		95% CI
Weather						
Mean temperature (°C)	1.05		1.02–1.09	NT		
Minimum temperature (°C)	1.06		1.02–1.10	1.06	(1.02–1.10)	
Maximum temperature (°C)	1.04		1.01–1.07	NT		
Precipitation (10 mm)	1.00		0.99–1.01	NA		
Age group (years)						
0–4	0.06		0.01–0.19	0.06	0.01–0.19	
5–17	0.54		0.38–0.75	0.52	0.37–0.72	
18–39	0.67		0.52–0.88	0.68	0.53–0.87	
40–59	Reference					
≥ 60	0.70		0.53–0.92	0.72	0.55–0.93	
Sex						
Female	Reference					
Male	5.78	4.45–7.63		5.82	4.48–7.67	

CI: confidence interval; IRR: incidence rate ratio; NA: not applicable; NT: not tested.

^a^ The multivariable negative binomial regression model was adjusted for whether a case was infected in a COVID-19 pandemic year of 2020 or 2021.

In multivariable negative binomial regression analysis, minimum temperature (IRR = 1.06; 95% CI: 1.02–1.10) and male sex (IRR = 5.86; 95% CI: 4.51–7.74) were significantly associated with increased weekly leptospirosis incidence (Table 3). Incidences were significantly lower among those aged 0–4 years (IRR = 0.06; 95% CI: 0.01–0.19), 5–17 years (IRR = 0.53; 95% CI: 0.38–0.73), 18–39 years (IRR = 0.67; 95% CI: 0.52–0.86) and ≥ 60 years (IRR = 0.72; 95% CI: 0.55–0.94) than in persons aged 40–59 years.

### Sensitivity analyses

In sensitivity analyses, no significant interactions were present between summer temperature (June–September) and precipitation (p > 0.05), winter temperature (December–February) and precipitation (p > 0.05), age and sex (p > 0.05), age and summer temperature (p > 0.05), age and winter temperature (p > 0.05), age and precipitation (p > 0.05), sex and summer temperature (p > 0.05), sex and winter temperature (p > 0.05) and sex and precipitation (p > 0.05).

## Discussion

From 2005 to 2023, the yearly number of notified leptospirosis cases in the Netherlands increased, with the largest increase in autochthonous cases. From 2005 to 2013, an average of 35 leptospirosis cases were notified annually, with a sudden increase to 90 cases in 2014. After a gradual decrease in the following years, the number of cases increased again in 2019 and has remained at his level, although the number of imported cases was lower in the COVID-19 pandemic years of 2020 and 2021. The annual incidence of autochthonous leptospirosis cases was significantly associated with higher temperatures during summer and the preceding winter. Higher temperatures between June and October were also associated with an increased weekly incidence of autochthonous leptospirosis cases. There was no association between precipitation and leptospirosis incidence.

In the Netherlands, rapid diagnostic tests, referring to a lateral flow immunochromatographic test for *Leptospira*-specific IgM antibody measurements [21], have been included as diagnostic tests in the notification criteria for leptospirosis since 2018. Although only a few cases have been notified based on a positive rapid diagnostic test only, pre-screening with a rapid diagnostic test may have led to more confirmative tests, like MAT, ELISA and/or PCR. Moreover, an increase in molecular testing may have also led to an increase in the number of detections. In contrast, however, the proportion of hospitalised cases increased from 2005 to 2023, which may suggest that testing is concentrated on hospitalised patients. The increase in autochthonous cases is more pronounced than the number of travel-related cases. Because changes in diagnostic practices would be expected to equally affect the number of diagnosed autochthonous and travel-related cases, this could indicate that at least the number of cases that acquired the infection in the Netherlands has increased over time. Altogether, we cannot rule out that changes in diagnostic testing could explain the increased leptospirosis incidence.

Contaminated surface water and soil have been the main sources of infection since 2014. Like other European countries [12], in the Netherlands, popularity of water-based recreational activities might have increased 2013–2021, as indicated in a Dutch water sports survey [22]. The number of imported cases has increased over time, with over half of the infections acquired in South-eastern Asia, mainly Thailand. However, as data on trends in the leptospirosis incidence in South-eastern Asia are inconclusive and data on travel destinations not (publicly) available, it is unclear whether this increase can be attributed to changes in travel destination and behaviour or an increase in the presence of *Leptospira* in the countries of destination.

Male sex was the most significant factor associated with leptospirosis. This is a well-known risk factor [23] and is likely due to knowledge, attitude and behaviour increasing exposure among males to *Leptospira* [24,25]. Information about leptospirosis risks and safe practices during recreational water activities may decrease the number of persons infected with *Leptospira*, not just within the Netherlands, but also among travellers, mainly those visiting South-eastern Asia. Furthermore, for workers in high-risk occupations, like farming, sewage management and water-related activities, stronger safety regulations are needed to ensure that workers have access to and use protective gear to reduce exposure to contaminated water and soil.

An increase in leptospirosis incidence was associated with higher mean summer temperatures and with higher mean temperatures during the preceding winter. Moreover, results indicate that the weekly leptospirosis incidence was associated with higher temperatures June–October. This is in line with previous studies in tropical climates reporting increased leptospirosis incidence with higher temperatures [6,26-28]. This might in part be explained by the prolonged survival of *Leptospira* in surface water and soil at higher temperatures, with an optimal growth temperature of 28–30°C in vitro [29], but in vivo data on the effect of temperature are lacking [30]. Furthermore, *Leptospira* may survive up to 1–2 months in water-soaked soils and up to 6 h in natural water sources [28]. In addition, people are more likely to be exposed to *Leptospira* at higher temperatures as they more often take part in water-based activities. Higher winter temperature might increase rat populations, the most important source of *Leptospira* in the Netherlands. Although the rat population in non-urban areas in the Netherlands 2014–2018 remained unchanged, the population size fluctuated [31]. However, food availability and rodent control may have more influence on the population than climatic factors.

We did not find an association between precipitation and incidence of human leptospirosis, in contrast to several studies that observed an increased leptospirosis incidence with more precipitation, although these studies were performed in tropical climates [6-8,26-28]. Additionally, some studies in tropical climates showed a correlation between flooding due to heavy rainfall and leptospirosis [32].

This study has several limitations. First, the notified leptospirosis incidence is an underestimation of the real incidence of leptospirosis in the Netherlands. The current surveillance system mostly captures the most severe cases that are hospitalised, while the majority of *Leptospira* infections in humans result in mild and self-limiting symptoms. This under-ascertainment could be more pronounced for autochthonous cases than for imported cases, since they were more often hospitalised. Improving clinical awareness about leptospirosis is crucial for timely and adequate treatment. Secondly, it was sometimes challenging to identify the most likely source of infection, as this information was sometimes missing, or multiple likely sources were recorded. This is reflected by one in five leptospirosis cases having an unknown source of infection. Third, we used weather data from a weather station in the central part of the Netherlands, while weather may vary somewhat across the country. However, this likely had limited influence on our results, as the Netherlands is a relatively small country without major natural barriers and climate differences; thus, temperature and precipitation follow the same patterns countrywide. Lastly, the association between weather and leptospirosis incidence is complex to study, as many factors, such as human behaviour, animal population, and survival of *Leptospira* play a role and are likely correlated. Different *Leptospira* reservoir populations may also react differently to weather change, although most autochthonous infections in the Netherlands are caused by serogroup Icterohaemorrhagiae with rats as reservoir [33]. In the current study design, it is not possible to assess which of these factors explains the relationship between weather and leptospirosis.

## Conclusion

We identified a positive association between higher temperatures and an increasing human leptospirosis incidence, meaning that the leptospirosis incidence could increase even further due to climate change. Most cases were hospitalised, highlighting underdiagnosis of less severe cases and the need to increase awareness about leptospirosis among clinicians. Surveillance could be improved by collecting data on the type and number of diagnostic leptospirosis tests performed by laboratories, in order to quantify the effect of changes in diagnostics practices on the number of notified leptospirosis case.

## Supplementary Data

398000 Supplementary Material
