# Supplementary material for: Increased incidence of human leptospirosis and the effect of temperature and precipitation, the Netherlands, 2005 to 2023
Source: Euro Surveill. 2025 Apr 17;30(15):2400611. doi: 10.2807/1560-7917.ES.2025.30.15.2400611 (PMC12007398; doi:10.2807/1560-7917.ES.2025.30.15.2400611)
Supplement: Supplementary Material [file 24-00611_PIJNACKER_Supplement.pdf]

Supplementary material to "Increased incidence of human leptospirosis and the effect of temperature and precipitation, the Netherlands, 2005 to 2023"

This supplementary material is hosted by Eurosurveillance as supporting information alongside the article "Increased incidence of human leptospirosis and the effect of temperature and precipitation, the Netherlands, 2005 to 2023", on behalf of the authors, who remain responsible for the accuracy and appropriateness of the content. The same standards for ethics, copyright, attributions and permissions as for the article apply. Supplements are not edited by Eurosurveillance and the journal is not responsible for the maintenance of any links or email addresses provided therein

Supplementary Table 1. *Pearson correlation coefficients of the correlation between the number of weekly leptospirosis cases and lagged weather variables in the summer (June-October)*

	Minimum temperature	Mean temperature	Maximum temperature	Precipitation
No lag	0.005	0.002	0.004	0.01
Lag 1 week	0.03	0.03	0.03	0.01
Lag 2 weeks	0.04	0.04	0.04	0.02
Lag 3 weeks	0.06	0.05	0.04	0.01
Lag 4 weeks	0.06	0.05	0.04	-0.02

Supplementary Table 1*. Correlation matrix of weather variables on year level*

	Mean minimum temperature winter	Mean temperature winter	Minimum temperature winter	Mean temperature year	Mean temperature summer	Cumulative precipitation year	Cumulative precipitation June-October	Cumulative precipitation June-September
Mean minimum temperature winter	1.00	0.95	0.88	0.69	0.28	0.22	-0.18	-0.03
Mean temperature winter	0.95	1.00	0.89	0.66	0.15	0.28	-0.09	0.07
Minimum temperature winter	0.88	0.89	1.00	0.57	0.22	0.31	-0.05	0.02
Mean temperature year	0.69	0.66	0.57	1.00	0.52	0.23	-0.23	-0.10
Mean temperature summer	0.28	0.15	0.22	0.52	1.00	-0.04	-0.43	-0.69
Cumulative precipitation year	0.22	0.28	0.31	0.23	-0.04	1.00	0.77	0.54
Cumulative precipitation June-October	-0.18	-0.09	-0.05	-0.23	-0.43	0.77	1.00	0.81
Cumulative precipitation June-September	-0.03	0.07	0.02	-0.10	-0.69	0.54	0.81	1.00

Supplementary Table 2. *Correlation matrix of weather variables on week level in the summer (June-October)*

	Mean temperature	Minimum temperature	Maximum temperature	Precipitation
Mean temperature	1.00	0.92	0.97	-0.13
Minimum temperature	0.92	1.00	0.82	0.06
Maximum temperature	0.97	0.82	1.00	-0.22
Precipitation	-0.13	0.06	-0.22	1.00

Supplementary Table 4. *Characteristics of autochthonous notified leptospirosis cases overall and by year, the Netherlands, 2005-2013*

	Total	2005	2006	2007	2008	2009	2010	2011	2012	2013	2014	2015	2016	2017	2018	2019	2020	2021	2022	2023	P-value[1]
	N = 596	N = 13	N = 12	N = 22	N = 11	N = 11	N = 14	N = 10	N = 21	N = 9	N = 58	N = 44	N = 35	N = 31	N = 20	N = 57	N = 56	N = 49	N = 49	N = 74	
	n (%)	n (%)	n (%)	n (%)	n (%)	n (%)	n (%)	n (%)	n (%)	n (%)	n (%)	n (%)	n (%)	n (%)	n (%)	n (%)	n (%)	n (%)	n (%)	n (%)	
Age category																					0.08
0-4 years	2 (0)	0 (0)	0 (0)	0 (0)	0 (0)	0 (0)	0 (0)	0 (0)	0 (0)	0 (0)	0 (0)	0 (0)	0 (0)	1 (3)	0 (0)	0 (0)	0 (0)	0 (0)	1 (2.0)	0 (0)	
5-17 years	64 (11)	2 (15)	1 (8)	2 (9)	2 (18)	1 (9)	0 (0)	1 (10)	3 (14)	3 (33)	4 (7)	9 (21)	4 (11)	2 (7)	2 (10)	8 (14)	11 (20)	3 (6)	1 (2.0)	5 (6.8)	
18-39 years	156 (26)	3 (23)	3 (25)	6 (27)	3 (27)	4 (36)	4 (29)	4 (40)	4 (19)	1 (11)	13 (22)	12 (27)	7 (20)	10 (32)	6 (30)	20 (35)	11 (20)	12 (25)	16 (33)	17 (23)	
40-59 years	232 (39)	4 (31)	7 (58)	8 (36)	4 (36)	4 (36)	5 (36)	4 (40)	12 (57)	2 (22)	28 (48)	13 (30)	15 (43)	10 (32)	9 (45)	16 (28)	20 (36)	23 (47)	15 (31)	33 (45)	
60 years and older	141 (24)	4 (31)	1 (8)	5 (23)	2 (18)	2 (18)	5 (36)	1 (10)	2 (10)	3 (33)	13 (22)	10 (23)	9 (26)	8 (26)	3 (15)	13 (23)	14 (25)	11 (22)	16 (33)	19 (26)	
Unknown	1 (0)	0 (0)	0 (0)	1 (4.5)	0 (0)	0 (0)	0 (0)	0 (0)	0 (0)	0 (0)	0 (0)	0 (0)	0 (0)	0 (0)	0 (0)	0 (0)	0 (0)	0 (0)	0 (0)	0 (0)	
Sex																					0.15
Female	93 (16)	1 (8)	1 (8)	2 (9)	0 (0)	1 (9)	0 (0)	1 (10)	4 (19)	1 (11)	12 (21)	10 (23)	6 (17)	6 (19)	2 (10)	7 (12)	6 (11)	8 (16)	12 (25)	13 (18)	
Male	502 (84)	12 (92)	11 (92)	20 (91)	11 (100)	10 (91)	14 (100)	9 (90)	17 (81)	8 (89)	46 (79)	34 (77)	28 (80)	25 (81)	18 (90)	50 (88)	50 (89)	41 (84)	37 (76)	61 (82)	
Unknown	1 (0)	0 (0)	0 (0)	0 (0)	0 (0)	0 (0)	0 (0)	0 (0)	0 (0)	0 (0)	0 (0)	0 (0)	1 (3)	0 (0)	0 (0)	0 (0)	0 (0)	0 (0)	0 (0)	0 (0)	
Diagnostic test[2]																					0.02
PCR and/or culture	228 (38)	0 (0)	2 (17)	2 (9)	1 (9)	2 (18)	2 (14)	0 (0)	4 (19.0)	6 (67)	25 (43)	15 (34)	15 (43)	17 (55)	13 (65)	17 (30)	25 (45)	18 (37)	23 (47)	41 (55)	
Serology	235 (39)	10 (77)	9 (75)	17 (77)	9 (82)	7 (64)	12 (86)	8 (80)	15 (71)	3 (33)	28 (48)	19 (43)	16 (46)	11 (36)	5 (25)	16 (28)	11 (20)	16 (33)	12 (25)	11 (15)	
Rapid diagnostic/lateral flow test	35 (5.9)	0 (0)	0 (0)	0 (0)	0 (0)	0 (0)	0 (0)	0 (0)	0 (0)	0 (0)	0 (0)	0 (0)	0 (0)	0 (0)	1 (5)	12 (21)	9 (16)	4 (8)	3 (6)	6 (8)	
Multiple tests	65 (11)	2 (15)	0 (0)	2 (9)	1 (9)	2 (18)	0 (0)	2 (20)	1 (5)	0 (0)	3 (5)	4 (9)	3 (9)	2 (7)	0 (0)	10 (18)	5 (9)	8 (16)	9 (18)	11 (15)	
Unknown	228 (38)	0 (0)	2 (17)	2 (9)	1 (9)	2 (18)	2 (14)	0 (0)	4 (19)	6 (67)	25 (43)	15 (34)	15 (46)	17 (55)	13 (65)	17 (30)	25 (45)	18 (37)	23 (47)	41 (55)	
Hospital admission																					0.03
Yes	511 (89)	11 (85)	7 (58)	21 (96)	9 (82)	9 (82)	12 (86)	10 (100)	17 (81)	3 (33)	54 (93)	28 (64)	30 (86)	29 (94)	16 (80)	48 (84)	51 (91)	43 (88)	46 (94)	67 (91)	
No	63 (11)	1 (8)	5 (42)	1 (5)	2 (18)	1 (9)	2 (14)	0 (0)	4 (19)	0 (0)	4 (7)	11 (25)	4 (11)	1 (3)	3 (15)	8 (14)	4 (7)	5 (10)	3 (6)	4 (5)	
Unknown	22 (4)	1 (8)	0 (0)	0 (0)	0 (0)	1 (9)	0 (0)	0 (0)	0 (0)	6 (67)	0 (0)	5 (11)	1 (3)	1 (3)	1 (5)	1 (2)	1 (2)	1 (2.0)	0 (0)	3 (4)	
Source of infection[3]																					0.91
Water/ soil	337 (71)	n/a	n/a	n/a	n/a	n/a	n/a	n/a	n/a	n/a	42 (72)	27 (61)	26 (74)	22 (71)	14 (70)	46 (81)	41 (73)	28 (57)	34 (69)	57 (77)	

Animal	49 (10)	n/a	n/a	n/a	n/a	n/a	n/a	n/a	n/a	n/a	7 (12)	5 (11)	6 (17)	2 (7)	2 (10)	3 (5)	2 (4)	6 (12)	10 (20)	6 (8)	
Other	1 (0)	n/a	n/a	n/a	n/a	n/a	n/a	n/a	n/a	n/a	0 (0)	0 (0)	0 (0)	0 (0)	0 (0)	0 (0)	0 (0)	1 (2.0)	0 (0)	0 (0)	
Unknown	86 (18)	n/a	n/a	n/a	n/a	n/a	n/a	n/a	n/a	n/a	9 (16)	12 (27)	3 (8.6)	7 (22.6	4 (20)	8 (14)	13 (23)	14 (29)	5 (10)	11 (15)	
Occupational infection[3]																					0.87
Yes	90 (19)	n/a	n/a	n/a	n/a	n/a	n/a	n/a	n/a	n/a	9 (16)	15 (34)	5 (14)	3 (10)	3 (15)	12 (21)	7 (13)	14 (29)	7 (14)	15 (20)	
No	323 (68)	n/a	n/a	n/a	n/a	n/a	n/a	n/a	n/a	n/a	41 (71)	23 (52)	25 (71)	23 (74)	17 (85)	37 (65)	39 (70)	29 (59)	36 (74)	53 (72)	
Unknown	60 (13)	n/a	n/a	n/a	n/a	n/a	n/a	n/a	n/a	n/a	8 (14)	6 (14)	5 (14)	5 (16)	0 (0)	8 (14)	10 (18)	6 (12)	6 (12)	6 (8)	

Abbreviations: PCR = polymerase chain reaction; n/a = not applicable

1: P-values are based on the Jonckheere-Terpstra test for trend excluding cases with an unknown for the variable in question.

2: Rapid diagnostic tests have been included in the notification criteria since 2018, before then cases notified by rapid diagnostic tests only could not be notified.

3: Source of infection and occupational infection were only summarized for cases notified from 2014 to 2023 (n = 473), as this information was not available for cases before 2014.

Supplementary Table 5. *Characteristics of imported notified leptospirosis cases overall and by year, the Netherlands, 2005-2013*

	Total	2005	2006	2007	2008	2009	2010	2011	2012	2013	2014	2015	2016	2017	2018	2019	2020	2021	2022	2023	P-value[1]
	N = 507<br>n (%)	N = 18<br>n (%)	N = 13<br>n (%)	N = 18<br>n (%)	N = 18<br>n (%)	N = 11<br>n (%)	N = 16<br>n (%)	N = 18<br>n (%)	N = 25<br>n (%)	N = 18<br>n (%)	N = 30<br>n (%)	N = 44<br>n (%)	N = 49<br>n (%)	N = 39<br>n (%)	N = 28<br>n (%)	N = 62<br>n (%)	N = 7<br>n (%)	N = 9<br>n (%)	N = 40<br>n (%)	N = 44<br>n (%)	
Age category																					0.13
0-4 years	0 (0)	0 (0)	0 (0)	0 (0)	0 (0)	0 (0)	0 (0)	0 (0)	0 (0)	0 (0)	0 (0)	0 (0)	0 (0)	0 (0)	0 (0)	0 (0)	0 (0)	0 (0)	0 (0)	0 (0)	
5-17 years	56 (11)	0 (0)	0 (0)	1 (6)	3 (17)	1 (9)	1 (6)	0 (0)	3 (12)	1 (6)	4 (13)	3 (7)	7 (14)	4 (10)	2 (7)	9 (15)	0 (0)	0 (0)	8 (20)	9 (21)	
18-39 years	289 (57)	13 (72)	8 (62)	12 (67)	10 (56)	8 (73)	4 (25)	12 (67)	12 (48)	9 (50)	18 (60)	27 (61)	28 (57)	29 (74)	16 (57)	35 (57)	4 (57)	2 (22)	20 (50)	22 (50)	
40-59 years	129 (25)	5 (28)	3 (23)	5 (28)	5 (28)	1 (9)	11 (69)	5 (28)	6 (24)	6 (33)	6 (20)	10 (23)	12 (25)	6 (15)	7 (25)	14 (23)	1 (14)	6 (67)	10 (25)	10 (23)	
60 years and older	33 (7)	0 (0)	2 (15)	0 (0)	0 (0)	1 (9)	0 (0)	1 (6)	4 (16)	2 (11)	2 (67)	4 (9)	2 (4)	0 (0)	3 (11)	4 (7)	2 (29)	1 (11)	2 (5)	3 (7)	
Unknown	0 (0)	0 (0)	0 (0)	0 (0)	0 (0)	0 (0)	0 (0)	0 (0)	0 (0)	0 (0)	0 (0)	0 (0)	0 (0)	0 (0)	0 (0)	0 (0)	0 (0)	0 (0)	0 (0)	0 (0)	
Sex																					0.20
Female	118 (23)	4 (22)	2 (15)	3 (17)	3 (17)	3 (27)	4 (25)	3 (17)	6 (24)	4 (22)	6 (20)	13 (30)	8 (16)	9 (23)	8 (29)	13 (21)	1 (14)	4 (44)	13 (33)	11 (25)	
Male	389 (77)	14 (78)	11 (85)	15 (83)	15 (83)	8 (73)	12 (75)	15 (83)	19 (76)	14 (78)	24 (80)	31 (71)	41 (84)	30 (77)	20 (71)	49 (79)	6 (86)	5 (56)	27 (68)	33 (75)	
Unknown	0 (0)	0 (0)	0 (0)	0 (0)	0 (0)	0 (0)	0 (0)	0 (0)	0 (0)	0 (0)	0 (0)	0 (0)	0 (0)	0 (0)	0 (0)	0 (0)	0 (0)	0 (0)	0 (0)	0 (0)	
Diagnostic test[2]																					<0.01
PCR and/or culture	196 (39)	3 (17)	0 (0)	4 (22)	4 (22)	1 (9)	4 (25)	1 (6)	5 (20)	8 (44)	16 (53)	14 (32)	23 (47)	16 (41)	11 (39)	26 (42)	3 (43)	4 (44)	26 (65)	27 (61)	
Serology	239 (47)	13 (72)	11 (85)	11 (61)	14 (78)	10 (91)	12 (75)	11 (61)	19 (76)	10 (56)	10 (33)	26 (59)	23 (47)	18 (46)	12 (43)	22 (36)	2 (29)	1 (11)	7 (18)	7 (16)	
Rapid diagnostic/lateral flow test	13 (3)	0 (0)	0 (0)	0 (0)	0 (0)	0 (0)	0 (0)	0 (0)	0 (0)	0 (0)	0 (0)	0 (0)	0 (0)	0 (0)	2 (7)	2 (3)	1 (14)	0 (0)	4 (10)	4 (9)	
Multiple tests	33 (7)	2 (11)	0 (0)	2 (11)	0 (0)	0 (0)	0 (0)	3 (17)	1 (4)	0 (0)	2 (7)	2 (5)	2 (4)	0 (0)	2 (7)	6 (10)	1 (14)	2 (22)	3 (8)	5 (11)	
Unknown	26 (5)	0 (0)	2 (15)	1 (6)	0 (0)	0 (0)	0 (0)	3 (17)	0 (0)	0 (0)	2 (7)	2 (5)	1 (2)	5 (13)	1 (4)	6 (10)	0 (0)	2 (22)	0 (0)	1 (2)	
Hospital admission																					<0.01
Yes	335 (66)	14 (78)	10 (77)	14 (78)	16 (89)	6 (55)	13 (81)	15 (83)	21 (84)	0 (0)	23 (77)	28 (64)	36 (74)	24 (62)	13 (46)	36 (58)	5 (71)	7 (78)	23 (58)	31 (71)	
No	138 (27)	3 (17)	3 (23)	4 (22)	2 (11)	4 (36)	3 (19)	3 (17)	4 (16)	2 (11)	7 (23)	12 (27)	13 (27)	12 (31)	14 (50)	21 (34)	2 (29)	0 (0)	17 (43)	12 (27)	
Unknown	34 (7)	1 (6)	0 (0)	0 (0)	0 (0)	1 (9)	0 (0)	0 (0)	0 (0)	16 (89)	0 (0)	4 (9)	0 (0)	3 (8)	1 (4)	5 (8)	0 (0)	2 (22)	0 (0)	1 (2)	
Region of infection																					0.11
Southeast Asia	303 (60)	7 (39)	11 (85)	11 (61)	10 (56)	9 (82)	11 (69)	14 (78)	14 (56)	10 (56)	23 (77)	28 (64)	31 (63)	20 (51)	12 (43)	40 (65)	2 (29)	0 (0)	22 (55)	28 (64)	

Central America and Caribbean	79 (16)	3 (17)	0 (0)	1 (6)	1 (6)	1 (9)	1 (6)	2 (11)	5 (20)	4 (22)	5 (17)	7 (16)	8 (16)	9 (23)	8 (29)	10 (16)	1 (14)	2 (22)	6 (15)	5 (11)	
Europe	62 (12)	5 (28)	0 (0)	5 (28)	5 (28)	0 (0)	3 (19)	2 (11)	6 (24)	0 (0)	1 (3)	4 (9)	2 (4)	3 (8)	5 (18)	4 (7)	1 (14)	6 (67)	3 (8)	7 (16)	
South America	38 (8)	2 (11)	1 (8)	0 (0)	2 (11)	1 (9)	1 (6)	0 (0)	0 (0)	2 (11)	1 (3)	1 (2)	6 (12)	4 (10)	2 (7)	4 (7)	3 (43)	0 (0)	6 (15)	2 (5)	
Other	20 (4)	1 (6)	1 (8)	1 (6)	0 (0)	0 (0)	0 (0)	0 (0)	0 (0)	2 (11)	0 (0)	1 (2)	2 (4)	2 (5)	1 (4)	4 (7)	0 (0)	0 (0)	3 (8)	2 (5)	
Unknown	303 (60)	7 (39)	11 (85)	11 (61)	10 (56)	9 (82)	11 (69)	14 (78)	14 (56)	10 (56)	23 (77)	28 (64)	31 (63)	20 (51)	12 (43)	40 (65)	2 (29)	0 (0)	22 (55)	28 (64)	
Source of infection[3]																					0.33
Water/ soil	272 (77)	n/a	n/a	n/a	n/a	n/a	n/a	n/a	n/a	n/a	24 (80)	36 (82)	23 (47)	18 (46)	23 (82)	55 (89)	6 (86)	7 (78)	37 (93)	43 (98)	
Animal	3 (1)	n/a	n/a	n/a	n/a	n/a	n/a	n/a	n/a	n/a	0 (0)	0 (0)	1 (2)	1 (3)	1 (4)	0 (0)	0 (0)	0 (0)	0 (0)	0 (0)	
Other	0 (0)	n/a	n/a	n/a	n/a	n/a	n/a	n/a	n/a	n/a	0 (0)	0 (0)	0 (0)	0 (0)	0 (0)	0 (0)	0 (0)	0 (0)	0 (0)	0 (0)	
Unknown	77 (22)	n/a	n/a	n/a	n/a	n/a	n/a	n/a	n/a	n/a	6 (20)	8 (18)	25 (51)	20 (51)	4 (14)	7 (11)	1 (14)	2 (22)	3 (8)	1 (2)	
Occupational infection[3]																					0.22
Yes	8 (2)	n/a	n/a	n/a	n/a	n/a	n/a	n/a	n/a	n/a	0 (0)	1 (2)	0 (0)	2 (5)	0 (0)	1 (1.6)	0 (0)	0 (0)	3 (8)	1 (2)	
No	317 (90)	n/a	n/a	n/a	n/a	n/a	n/a	n/a	n/a	n/a	29 (97)	40 (91)	46 (94)	32 (82)	27 (96)	54 (87)	6 (86)	7 (78)	37 (93)	39 (89)	
Unknown	27 (8)	n/a	n/a	n/a	n/a	n/a	n/a	n/a	n/a	n/a	1 (3)	3 (7)	3 (6)	5 (13)	1 (4)	7 (11)	1 (14)	2 (22)	0 (0)	4 (9)	

Abbreviations: PCR = polymerase chain reaction; n/a = not applicable

1: P-values are based on the Jonckheere-Terpstra test for trend excluding cases with an unknown for the variable in question.

2: Rapid diagnostic tests have been included in the notification cri2teria since 2018, before then cases notified by rapid diagnostic tests only could not be notified.

3: Source of infection and occupational infection were only summarized for cases notified from 2014 to 2023 (n = 842), as this information was not available for cases before 2014.

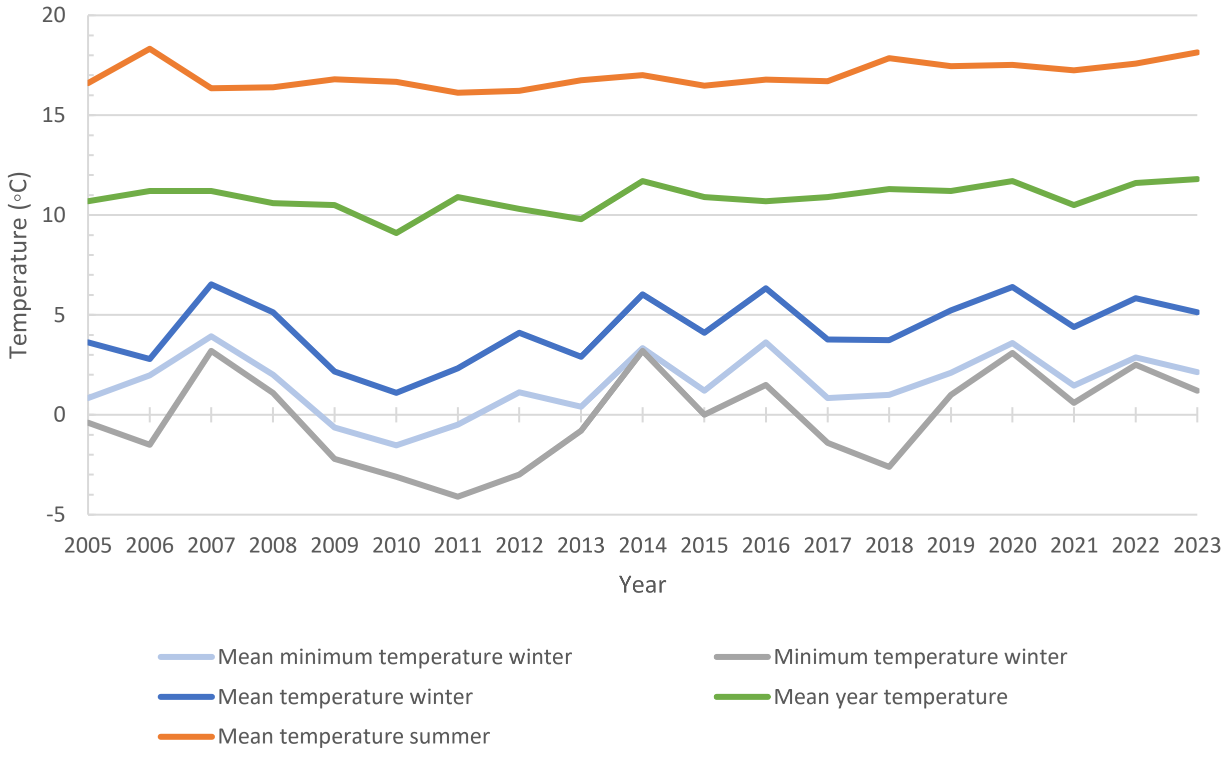

Supplementary Figure 1. *Variables on winter, summer, and year temperature in the Netherlands 2005-2023*

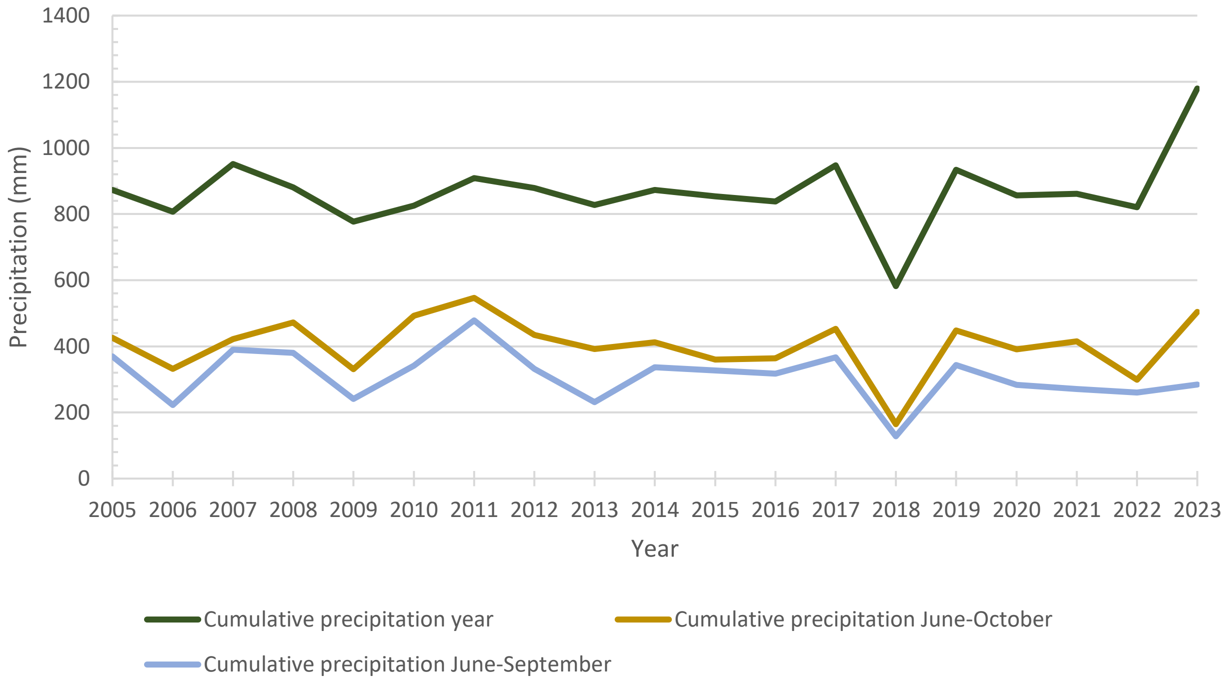

Supplementary Figure 2. *Variables on cumulative precipitation in the summer and per year in the Netherlands 2005-2023*
